# Ultrasound-Guided Percutaneous Peripheral Nerve Stimulation of the Musculocutaneous Nerve for Refractory Antecubital ElbowPain—Brief Technical Report and Illustrative Case Report

**DOI:** 10.1080/24740527.2023.2249054

**Published:** 2023-08-22

**Authors:** Quinn Tate, Guilherme Ferreira-Dos-Santos, Darrell Vydra, Nuno Ferreira-Silva, Sahil Gupta, Mark Friedrich B. Hurdle

**Affiliations:** a Department of Physical Medicine and Rehabilitation, Penn Medicine, University of Pennsylvania, Philadelphia, Pennsylvania, USA; b Division of Pain Medicine, Department of Anesthesiology, Reanimation, and Pain Medicine, Hospital Clínic de Barcelona, University of Barcelona, Barcelona, Catalonia, Spain; c Department of Pain Medicine, Mayo Clinic, Jacksonville, Florida, USA; d Department of Physical Medicine and Rehabilitation, Hospital Professor Doutor Fernando Fonseca, Amadora, Portugal

**Keywords:** Pain medicine, neuromodulation, peripheral neuropathy, peripheral nerve stimulation, ultrasound

## Abstract

Chronic pain following distal biceps rupture (DBR) is often nonspecific in that it may arise due to the injury, subsequent surgical repair, or a combination of factors, making the painful symptoms challenging to treat. Peripheral nerve injury in the setting of DBR most commonly affects the musculocutaneous nerve or one of its terminal branches and may lead to chronic neuropathic pain involving the elbow and lateral/radial aspect of the forearm. In this brief technical report, we describe an ultrasound-guided (USG) technique for percutaneous implantation of a peripheral nerve stimulator (PNS) targeting the musculocutaneous nerve, along with an illustrative case report of successful treatment of chronic refractory pain following DBR utilizing this technique. Six months postimplantation, the patient reported a greater than 60% baseline pain intensity reduction, and no complications were noted.

## RÉSUMÉ

La douleur chronique consécutive à une rupture distale du biceps est souvent non spécifique en ce sens qu’elle peut survenir en raison de la blessure, de la réparation chirurgicale ultérieure, ou d’une combinaison de facteurs, rendant les symptômes douloureux difficiles à traiter. La lésion du nerf périphérique dans le cadre d’une rupture distale du biceps affecte le plus souvent le nerf musculo-cutané ou l’une de ses branches terminales et peut entraîner des douleurs neuropathiques chroniques au niveau du coude et de la face latérale/radiale de l’avant-bras. Dans ce bref rapport technique, nous décrivons une technique guidée par ultrason pour l’implantation percutanée d’un stimulateur nerveux périphérique ciblant le nerf musculo-cutané, ainsi qu’un rapport de cas illustratif du traitement réussi de la douleur réfractaire suite à une rupture distale du biceps en utilisant cette technique. Six mois après l’implantation, le patient a signalé une réduction de plus de 60 % de l’intensité de la douleur initiale, et aucune complication n’a été observée.

## Introduction

Chronic, intractable, antecubital elbow pain can occur following distal biceps brachii rupture (DBR) and surgical repair, with injury to the lateral antebrachial cutaneous nerve (LACN) being the most common complication at follow-up.^1–4^ Surgical repair is the current standard of care to minimize strength loss and fatigue, with outcomes for surgery delayed by 6 weeks and increasing complication rates.^5^ Up to 10% of patients with sensory peripheral nerve injury following surgical biceps tendon repair reported chronic pain at follow-up.^1^ Grewal et al.^6^ hypothesized that entrapment of the LACN in postoperative scar tissue could be responsible for postsurgical pain in this group of patients, especially when pain has a neuropathic character.^3^

**Figure 1.**
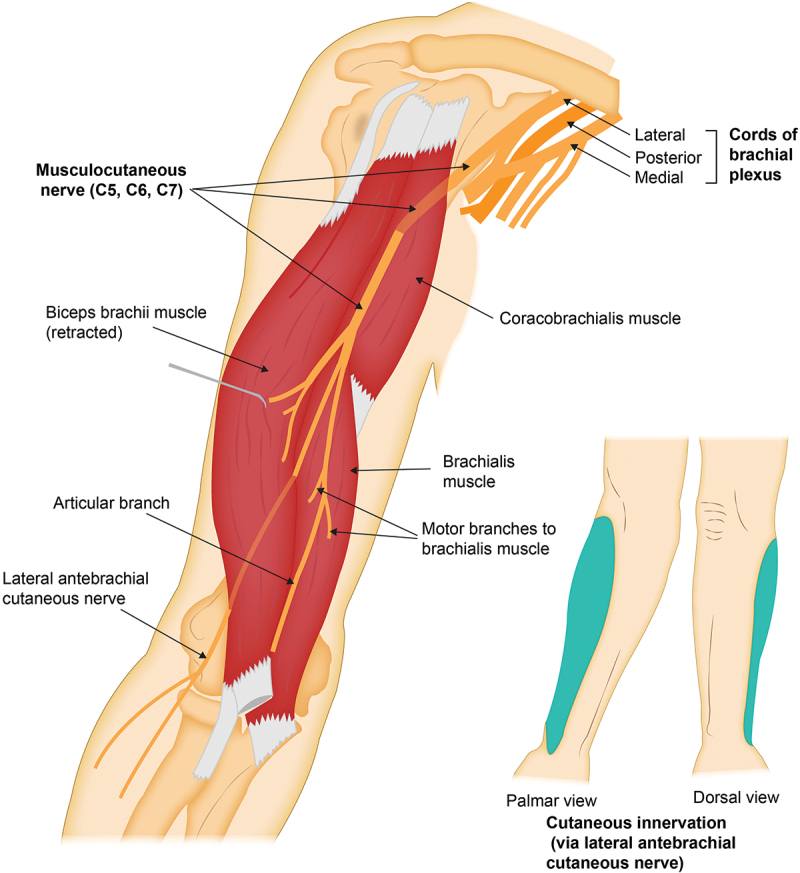
Origin and trajectory of the musculocutaneous nerve from the lateral cord of the brachial plexus to the lateral (radial) aspect of the forearm, with illustration of the main motor branches and cutaneous area of innervation in the lateral (radial) forearm (via its terminal branch, the lateral antebrachial cutaneous nerve).

The musculocutaneous nerve (MCN) is a terminal branch of the lateral cord of the brachial plexus, with contributions from the fifth, sixth, and seventh cervical nerve roots (C5, C6, and C7). The MCN is responsible for motor innervation to the coracobrachialis, biceps brachii, and brachialis, and its terminal branch, the LACN, provides cutaneous innervation to the lateral (radial) forearm.^7^ The MCN branches off the lateral cord of the brachial plexus about 5.5 to 6 cm distal to the origin of the coracobrachialis, at which point it pierces the deep surface of the muscle within the axilla.^6,7^ The MCN then exits the coracobrachialis and traverses within the anterior compartment of the arm, deep to the biceps brachii and superficial to the brachialis. At this point, it gives off motor branches to the biceps brachii and the brachialis (on average at 13.0 and 17.5 cm, respectively, as measured from the acromion process).^7^ From this point distally, the MCN does not provide any motor innervation, continuing between the biceps brachii and the brachialis until it exits the interfascial muscle plane just lateral to the biceps tendon, about 2 cm proximal to the antecubital fossa.^7,8^ Once it leaves this compartment, it becomes the LACN and continues distally providing volar and dorsal cutaneous innervation along the lateral (radial) forearm (Figure 1).^8^

Over the last 20 years, electrical neuromodulation techniques have reemerged as a viable minimally invasive approach to the treatment of medically refractory neuropathic pain, having outshined other available procedures in the last 10 years.^9–11^ Among the different types of available neuromodulation techniques, ultrasound-guided (USG) percutaneous implantation of a peripheral nerve stimulator (PNS) is minimally invasive, although it is also the least established in terms of scientific evidence and regulatory approvals. Over the last 10 years, it has been gaining momentum in terms of the development of new indications and the accumulation of clinical experience.^9–11^

Several authors have hypothesized that pain relief from PNS, as sensed through paresthesia, is mediated by orthodromic stimulation of nonnociceptive Aβ fibers present in the free nerve endings of the peripheral nervous system. This stimulation subsequently leads to the activation of the inhibitory interneurons that are involved in the processing and transmission of nociceptive information by peripheral Aδ and C nerve fibers in the superficial layers of the dorsal horn of the spinal cord.^11^

USG techniques for percutaneous implantation of PNS targeting the median, ulnar, and radial nerves have previously been reported in the literature.^8^ Although members of our group have described distal targeting of the LACN, to the best of our knowledge, no USG technique has been reported for percutaneous implantation of a PNS targeting the MCN proximal to the antecubital fossa.^11,12^

In this technical report, we aimed to describe a USG technique for percutaneous implantation of a PNS targeting the MCN proximal to the antecubital fossa. We also present an illustrative case report of a patient with previously refractory, neuropathic, postsurgical antecubital elbow pain in whom implantation of the PNS along the MCN provided clinically significant analgesic benefit.

## Case Report

A 40-year-old male presented to our Department of Pain Medicine for assessment in the setting of moderate-to-severe pain and paresthesia in the antecubital region of the left elbow. He noted that his pain persisted following a work-related injury 2 years prior and subsequent surgical repair of the distal biceps tendon. At the time of injury, the patient was diagnosed with a high-grade partial-thickness tear of his left biceps tendon, and a surgical repair was performed without periprocedural complications. Following surgery, the patient completed a rehabilitation program, eventually regaining full active range of motion (ROM) and muscle strength in the elbow flexors and forearm supinators. However, over the months following surgery, he developed spontaneous episodes of severe, intermittent pain that he described as sharp, burning, and prickling, involving the left antecubital region, radiating distally along the forearm’s lateral (radial) aspect. He rated the pain as 9 on a 0 to 10 numeric rating scale (NRS). Although he experienced this described paresthesia, on exam he had normal sensation to light touch and pinprick testing. He did not have skin color changes or symptoms of hyperesthesia or allodynia. Radiographs of the elbow showed normal anatomic alignment without focal abnormalities. He completed 1 year of formal occupational therapy and physiotherapy, with a multimodal program that included active ROM exercises coupled with the eccentric strengthening of the elbow flexors and forearm supinators, as well as treatment with transcutaneous electrical nerve stimulation and iontophoresis, without noticeable improvement in his elbow pain. In addition, medical management with a combination of gabapentinoids, tricyclic antidepressants, and selective serotonin and norepinephrine reuptake inhibitors was unsuccessful in providing adequate and long-lasting pain relief despite adequate dosing. Because these medications did not provide any appreciable benefit, the patient stopped taking them.

The patient was eventually referred to our Department of Pain Medicine 2 years after his initial injury after failing the conservative treatments mentioned above. At our initial assessment, he presented with normal muscle strength in the elbow flexors and forearm supinators, no sensory deficits or abnormalities, and full passive and active ROM of the elbow. He described his pain as covering the entire antecubital region of the elbow, radiating distally along the lateral (radial) aspect of the forearm, generally dull and achy, with spontaneous episodes of severe, intermittent, sharp pain, with an intensity rated as 9 on a 0 to 10 NRS. These episodes were debilitating to the point of leaving him unable to continue to work. Furthermore, he reported bothersome paresthesia in the antecubital region of the elbow.

Given his normal sensory exam and no history of numbness following his surgery, a nerve conduction study of the LACN was not performed. Due to the distribution of pain, in association with the spontaneous episodes of severe, intermittent, sharp pain, a determination was made to perform a diagnostic USG block of the MCN at the level of the mid-humerus. This procedure resulted in over 90% pain relief and self-reported improvement in function for over 6 h, which is consistent with pain distal to the injection site, consistent with entrapment neuropathy or injury involving the distal MCN/LACN. Given these improvements following the block, the following technique was used for percutaneous stimulation.

## Technique Description

USG percutaneous implantation was performed in the Department of Pain Medicine setting at a tertiary academic medical institution in Jacksonville, Florida. The patient provided written consent authorizing the publication of the images shown for publication and medical education purposes. Ultrasound equipped with a 6- to 15-MHz linear array transducer was utilized for this technique.

**Figure 2.**
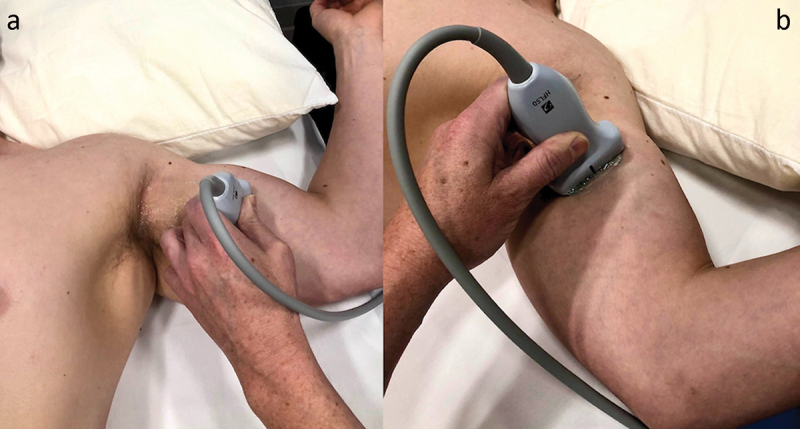
Patient position for the preprocedural ultrasound scan of the musculocutaneous nerve proximal to the antecubital fossa. (a) From an oblique angle view. (b) From an antero-posterior angle view. Note the position of the target upper extremity in 60° to 90° of shoulder abduction, 90° of shoulder external rotation, and 90° of elbow flexion. Note: The model shown in the photographs is a healthy volunteer. Consent was obtained from the volunteer for publication of these images.

**Figure 3.**
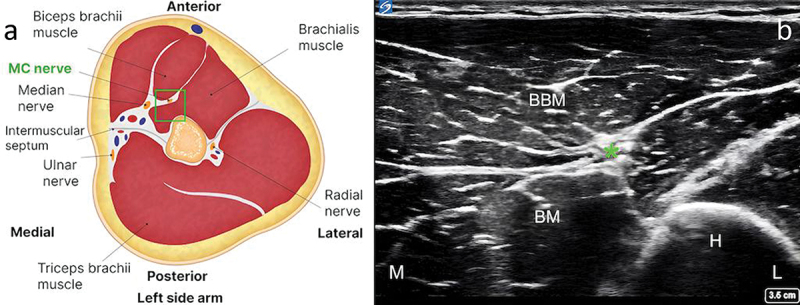
Identification of the musculocutaneous nerve in the interfascial muscle plane deep to the biceps brachii and superficial to the brachialis. (a) Anatomical illustration depicting the musculocutaneous nerve in the interfascial muscle plane deep to the biceps brachii and superficial to the brachialis (left side). (b) Corresponding ultrasound scan showing the musculocutaneous nerve and its relation to adjacent structures (right side). Note: The green square in the left-hand figure shows the area of interest, with the musculocutaneous nerve in the interfascial plane deep to the biceps brachii and superficial to the brachialis. BBM = biceps brachii muscle; BM = brachialis muscle; H = humerus; L = lateral; M = medial. *Musculocutaneous nerve.

The patient was positioned in the supine position, with the target upper extremity placed in 60° to 90° of shoulder abduction, 90° of shoulder external rotation, and 90° of elbow flexion (Figure 2). A preprocedural scan of the MCN was performed along the entire length of the nerve, starting in the axilla and continuing distally until the antecubital fossa. In our experience, an initial ultrasound assessment of the nerve is easier to perform with the transducer positioned to visualize the short axis of the nerve. This allows for optimal planning of the target site for needle entry to the skin and for the trajectory of lead implantation. According to our clinical experience, the optimal site for the final lead position is close to the mid-humerus, just distal to the MCN’s main motor branches to the brachialis.^7^ At this point, the nerve can be identified in the interfascial muscle plane deep to the biceps brachii and superficial to the brachialis muscles (Figure 3). The color Doppler mode may be utilized to note any vascular structures in the planned trajectory of lead implantation. If a local anesthetic is used before the implantation procedure, the volume of injectate and depth of injection should be carefully considered to reduce the chance of anesthetizing the MCN, thus potentially compromising sensory testing during implantation. In our clinical experience, the easiest way to implant an electrode along the MCN is using an in-plane approach from medial to lateral, at the level of the mid-humerus. This allows for easy repositioning of the electrode, either superficial or deep, to the nerve during the implantation procedure, which in turn maximizes the chances of achieving adequate sensory stimulation coverage of the painful area. An in-plane approach also minimizes inadvertent damage to vascular structures or the nerve. The optimal distance from the site of stimulator electrode implantation to the nerve should take into consideration the manufacturer’s instructions for the specific PNS system in use.

The decision was made to place a temporary percutaneously implanted PNS with USG targeting the MCN at the level of the mid-humerus. We decided to use temporary PNS (not a “permanent” PNS implant) due to the reported potential for long-term benefits^13^ and the lack of previous descriptions in the literature of implantation of permanent PNS systems targeting the MCN proximal to the antecubital fossa. During the procedure, a single PNS lead was adequately implanted parallel and superficial to the MCN at the level of the interfascial muscle plane between the biceps brachii and the brachialis. Full stimulation coverage of the antecubital region and lateral (radial) forearm was achieved at 46 mA at a frequency of 100 Hz.

During the month following placement of the temporary PNS system, the patient reported that his overall pain intensity decreased to an average of 2 on a 0 to 10 NRS, and he stopped having spontaneous episodes of severe, intermittent, sharp pain. In addition, during this period, he experienced resolution of the burning pain in the antecubital region of the elbow and was able to return to work 2 weeks following the procedure.

Four weeks following implantation, the patient reported that his pain had improved so dramatically that he increased his activity levels, despite recommendations to avoid heavy lifting, and experienced a sudden increase in pain following strenuous labor. Subsequent ultrasound examination revealed a migration of the implanted (temporary) lead. This was thought to be the most likely cause of adequate sensory stimulation coverage loss. A decision was made to remove the percutaneous lead during the clinic visit, and placement of a new temporary single lead system was planned. Placement of the new lead was successfully achieved following the technique described previously, and the final lead position was achieved near the same site, parallel and superficial to the MCN, at the level of the interfascial muscle plane between the biceps brachii and the brachialis. Following the second implantation procedure, the patient experienced complete resolution of pain and paresthesia over the antecubital region and lateral (radial) aspect of the forearm for the entirety of the 60-day temporary PNS placement.

**Figure 4.**
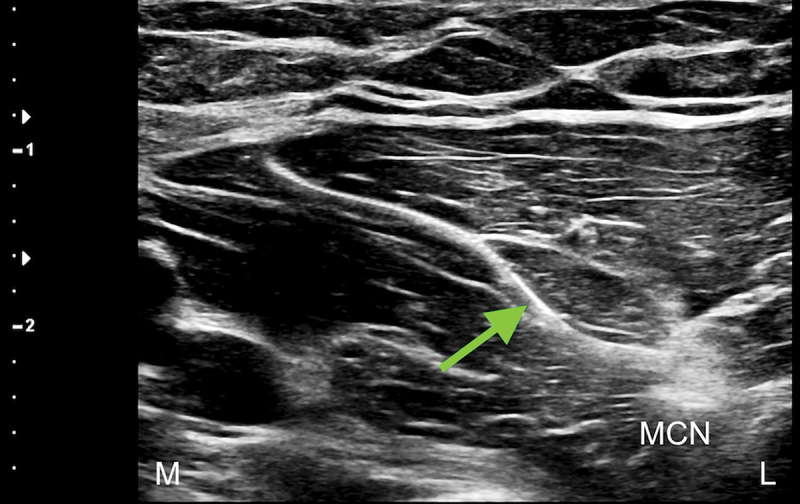
Ultrasound-guided medial-to-lateral in-plane percutaneous deployment of the peripheral nerve stimulator electrode, with visualization of the electrode tip just medial and superficial to the musculocutaneous nerve. Note: BBM = biceps brachii muscle; L = lateral; M = medial. Green arrow shows peripheral nerve stimulator electrode.

**Figure 5.**
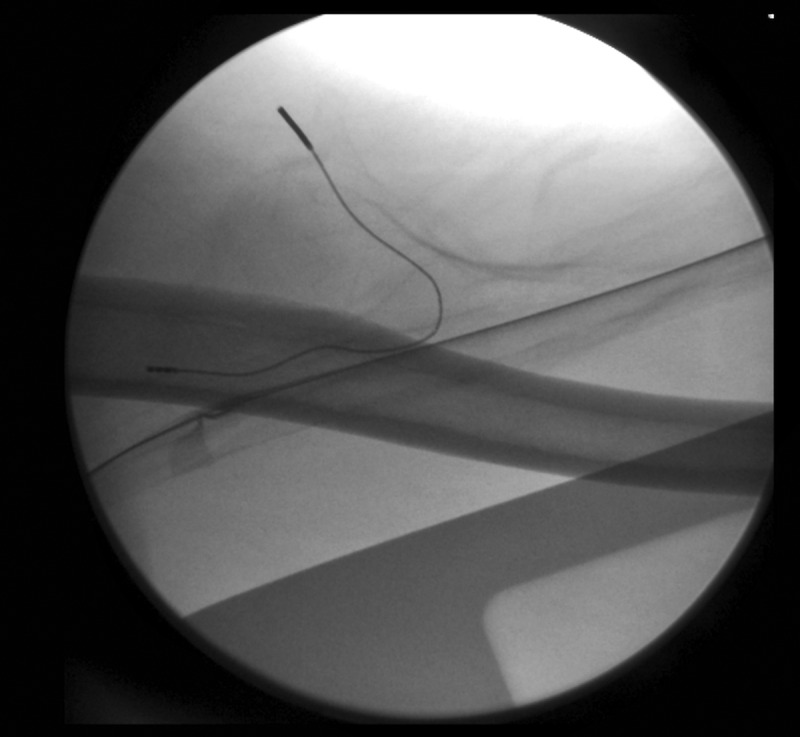
Fluoroscopic image used only to show final electrode position relative to the humeral shaft, at the level of the mid-humerus.

Over the weeks following the removal of the second temporary PNS, after completing the 60-day stimulation period, the patient reported a progressive return of pain in the left antecubital region, associated with a self-reported decrease in work performance during heavy labor tasks. When the patient was followed up 12 weeks post lead removal, his pain had returned to baseline. Taking into consideration the success of the previous treatment with a temporary PNS, the decision was made to perform a USG percutaneous implantation of a permanent PNS targeting the MCN at the level of the mid-humerus. During the procedure, the permanent lead was implanted parallel, at 0.5 cm medial, and superficial to the nerve (Figure 4). Full sensory stimulation coverage of the antecubital region and lateral (radial) forearm was achieved, and a fluoroscopic image was obtained only to demonstrate the final lead position relative to the humerus (Figure 5). The final programming of the permanent PNS was completed 14 days following implantation.

There were no procedure-related complications at 6 months postimplantation of the permanent PNS. At the time of writing this report, the patient reported that he was utilizing the device for approximately 4 h per day with the standard paresthesia-based stimulation protocol of the device set at a frequency of 100 Hz; a pulse width of 100 μs; an amplitude of 15 mA; a cycle of 50s on, 5 s off; and a 5-s ramp-up time. He has noted complete resolution of paresthesia and an overall average daily pain of 3 on a 0 to 10 NRS. Three weeks after the procedure, the patient returned to work without limitations and reported a significant improvement in his overall quality of life.

## Discussion

In the present study, a USG technique that allows for percutaneous implantation of a PNS targeting the MCN at the level of the mid-humerus, proximal to the antecubital fossa, was described and illustrated with a case report to demonstrate the feasibility of the procedure in the clinical setting. This technique was found to be a viable option for the implantation of both temporary and permanent PNS along the MCN to treat previously refractory, severe neuropathic pain involving the antecubital region of the elbow and the lateral (radial) aspect of the forearm. The patient’s description of painful paresthesia with the burning and prickling sensation is consistent with neuropathic pain. However, because he had a normal sensory exam with light touch and pinprick sensation, we did not utilize an electrodiagnostic study for evidence of nerve injury, although this would have been reasonable to obtain. His chronic pain symptoms did not improve for approximately 2 years after his DBR surgical repair. His pain did improve with stimulation and returned without active stimulation. However, we cannot be sure that his symptoms would not have spontaneously resolved without stimulation. Also, even though his symptoms did not follow a specific cervical nerve root dermatome, the possibility of a cervical radiculopathy cannot be ruled out. Given the focal symptoms and his response to targeted nerve blocks, we decided to focus treatment on the MCN. Because the MCN is the main supplier of cutaneous innervation to the antecubital region of the elbow and the volar and dorsal aspects of the lateral (radial) forearm, the clinical success of this USG technique targeting the MCN proximal to the elbow joint line could be in part due to the accessibility of the MCN as it courses in the interfascial muscle plane deep to the biceps brachii and superficial to the brachialis.^7^

From our clinical experience, several aspects of this technical approach are worth mentioning when analyzing its feasibility and reproducibility in the clinical setting. Firstly, by targeting the MCN at the level of the mid-humerus, distal to the emergence of the MCN’s major motor branches to the biceps brachii and the brachialis, this technical approach allows for access to the nerve under USG, while remaining proximal to the elbow joint line. This helps to ensure both adequate stimulation coverage of the antecubital region of the elbow and the lateral (radial) aspect of the forearm. In addition, it allows for a more straightforward execution of the implantation procedure compared to a more proximal approach closer to the axilla. Secondly, by targeting the nerve in the short axis through an in-plane approach from lateral to medial, this technique allows for easy positioning of the electrode near the MCN during the implantation procedure, thus maximizing the chances of achieving adequate sensory stimulation coverage of the painful area. Lastly, careful attention should be paid during the electrode deployment phase of the procedure to minimize the risk of “pull back” and loss of stimulation coverage when removing the electrode introducer. Consideration of placement to reduce repetitive changes in tension with regular muscle contraction is also important. Because this technique approaches the nerve in its short axis, even small amounts of “pull back” during the deployment phase may lead to loss of stimulation compared to implantation techniques targeting the nerve in its long axis.

In conclusion, the USG technique for percutaneous implantation of a PNS targeting the MCN at the level of the mid-humerus is a feasible approach to implantation of a percutaneous PNS along the MCN to treat previously refractory, severe neuropathic pain in the antecubital region of the elbow and lateral (radial) aspect of the forearm. Larger prospective case series and randomized controlled clinical trials are warranted to assess the clinical efficacy and long-term complications of USG percutaneous PNS of the MCN in the setting of chronic pain mediated by the musculocutaneous nerve.
